# *Lra*I from* Lactococcus raffinolactis* BGTRK10-1, an Isoschizomer of* Eco*RI, Exhibits Ion Concentration-Dependent Specific Star Activity

**DOI:** 10.1155/2018/5657085

**Published:** 2018-01-29

**Authors:** Marija Miljkovic, Milka Malesevic, Brankica Filipic, Goran Vukotic, Milan Kojic

**Affiliations:** ^1^Institute of Molecular Genetics and Genetic Engineering, University of Belgrade, Belgrade, Serbia; ^2^Faculty of Pharmacy, University of Belgrade, Belgrade, Serbia; ^3^Faculty of Biology, University of Belgrade, Belgrade, Serbia

## Abstract

Restriction enzymes are the main defence system against foreign DNA, in charge of preserving genome integrity.* Lactococcus raffinolactis* BGTRK10-1 expresses* Lra*I Type II restriction-modification enzyme, whose activity is similar to that shown for* Eco*RI;* Lra*I methyltransferase protects DNA from* Eco*RI cleavage. The gene encoding* Lra*I endonuclease was cloned and overexpressed in* E. coli*. Purified enzyme showed the highest specific activity at lower temperatures (between 13°C and 37°C) and was stable after storage at −20°C in 50% glycerol. The concentration of monovalent ions in the reaction buffer required for optimal activity of* Lra*I restriction enzyme was 100 mM or higher. The recognition and cleavage sequence for* Lra*I restriction enzyme was determined as 5′-G/AATTC-3′, indicating that* Lra*I restriction enzyme is an isoschizomer of* Eco*RI. In the reaction buffer with a lower salt concentration,* Lra*I exhibits star activity and specifically recognizes and cuts another alternative sequence 5′-A/AATTC-3′, leaving the same sticky ends on fragments as* Eco*RI, which makes them clonable into a linearized vector. Phylogenetic analysis based on sequence alignment pointed out the common origin of* Lra*I restriction-modification system with previously described* Eco*RI-like restriction-modification systems.

## 1. Introduction

Restriction endonucleases are generally accompanied by a cognate methyltransferase [1]. Both enzymes working together form a restriction-modification system (RM system). RM systems are important for the maintenance of the genome integrity of prokaryotic organisms. The range of biological processes that utilize RM system also includes involvement in DNA transposition [2] and recombination [3]. In addition, there is evidence that the genes for restriction and modification enzymes may act together as selfish elements [4, 5].

Restriction endonucleases exhibit high sequence specificity in substrate binding and use versatile DNA cleavage mechanisms and thus are excellent model systems for understanding DNA recognition and phosphodiester bond hydrolysis. Restriction endonucleases are classified according to their subunit composition, cofactor requirement, recognition site, cleavage site, and mode of action to define the different types (I, II, III, and IV). Restriction endonucleases Type II are essential tools for recombinant DNA technology. It seems unlikely that today's modern molecular biology and the biotechnology industry would have developed without Type II restriction enzymes. Because of their great importance in gene analysis and cloning there is a constant need to discover new ones. According to data from the REBASE [6, http://rebase.neb.com] which summarizes all information known about every restriction enzyme and any associated protein, there are more than 3945 biochemically or genetically characterized restriction enzymes and, out of 3834 Type II restriction enzymes, 299 distinct specificities are known. By 2010, six hundred and forty-one restriction enzymes were commercially available, including 235 distinct specificities [7]. Because of the large number of sequenced genomes, rate of discovery of new putative restriction and modification genes is rising rapidly. In contrast, the number of restriction enzymes that are biochemically characterized has actually dropped down to the level that was three decades ago.

Restriction endonucleases Type II are homodimeric or tetrameric enzymes that cleave DNA at defined sites of 4–8 bp in length and require Mg^2+^ ions for catalysis [8]. For many of restriction endonucleases Type II, it was found that modified conditions (lower ionic strength, higher pH, presence of different metallic cofactors, and organic solvents) could decrease their substrate specificity [9–13]. Under nonoptimal restriction conditions, these endonucleases can usually cleave degenerate sequences, which differ from standard recognition sites at only one nucleotide. This alteration in digestion specificity causing cleavage of DNA at novel, similar but not identical sequences is defined as enzyme star activity. Modified specificity of restriction enzyme (star) activity could be exploited to facilitate recombinant DNA techniques since the same enzyme in controlled conditions could recognize different DNA sequences and cleave at additional positions [9].

Restriction endonucleases with identical recognition sites isolated from different organisms are termed isoschizomers [7, 14]. The* Rsr*I endonuclease found in* Rhodobacter sphaeroides* is an isoschizomer of the* Eco*RI. Both enzymes recognize the sequence GAATTC and cleave it at the same position (G/AATTC) and are sharing 50% amino acid sequence identity [15]. Interesting,* Mun*I recognizes the sequence CAATTG, which differs from the recognition sequence of* Eco*RI (and* Rsr*I) only in the external base pairs. Comparison of the* Mun*I amino acid sequence with that of* EcoR*I and* Rsr*I revealed only a low level of overall similarity wherefore sequence homology between* Eco*RI and* Rsr*I has a stronger significance [16].

This work describes for the first time the occurrence of* Eco*RI-like restriction-modification genes in lactococci. The objective was to clone, purify, and biochemically and genetically characterize novel lactococcal* Lra*I restriction enzyme.* Lra*I restriction enzyme was overexpressed and purified to the homogeneity from* E. coli* using pMAL expression and purification system. Results demonstrate that* Lra*I restriction enzyme, although an isoschizomer of* Eco*RI, shows different characteristics. One of characteristics that could be further exploited is star activity of* Lra*I that is limited to one variant of the recognition site, which after cleavage leaves identical cohesive ends as* Eco*RI and* Lra*I restriction enzymes, so that the fragments obtained after digestion could be cloned without additional processing.

## 2. Material and Methods

### 2.1. Bacterial Strains and Culture Conditions

*Lactococcus raffinolactis* BGTRK10-1 was isolated from autochthonous sweet kajmak produced from sheep milk without the use of starter cultures in a household of the Vlašić mountain region, central Bosnia and Herzegovina [17] (Table 1). Preliminary strain classification was done according to its fermentation ability using API 50CHL (Api System SA; Bio-Merieux, Montelieu-Vercieu, France), temperature of growth (30°C, 37°C, and 45°C), growth in the presence of salt (4% and 6.5%), and pH tolerance. Final taxonomic classification of BGTRK10-1 was performed by sequencing of amplified 16S rDNA using primers previously described [18]. The strain was grown in M17 medium (Merck GmbH, Darmstadt, Germany) supplemented with D-glucose (0.5% w/v) (GM17) at 30°C.* Escherichia coli* DH5*α*, HB101, and ER2523 strains were grown aerobically in Luria-Bertani (LB) broth at 37°C, unless otherwise specified. Solid medium was made by adding 1.75% (w/v) agar (Torlak, Belgrade, Serbia), to the liquid media. Antibiotics were used at the following concentrations: erythromycin 300 *μ*g/ml and ampicillin 100 *μ*g/ml for selection and maintaining of transformants. The 5-bromo-4-chloro-3-indolyl-*β*-D-galacto-pyranoside (X-Gal) (Fermentas, Vilnius, Lithuania) was added to LB medium plates for blue/white colour screening of colonies with cloned fragments at final concentration of 40 *μ*g/ml.

### 2.2. Construction of Cosmid Library of* L. raffinolactis* BGTRK10-1

Total DNA isolated from the* L. raffinolactis* BGTRK10-1 was partially digested with* Xba*I restriction enzyme. Incubation was carried out during 1 h and was stopped in different time intervals by adding EDTA. Optimally digested DNA, giving fragments 30–40 kb, was purified and ligated overnight at 16°C with the pAZILcos vector [19] predigested with* Xba*I restriction enzyme and dephosphorylated. Ligation for formed concatemers of high molecular weight was checked on agarose gel and encapsulated into phage particles using packaging kit (Agilent Technologies). Encapsulated cosmids were transfected into* E. coli* HB101 magnesium cells and selection of clones was done on LA plates containing erythromycin 300 *μ*g/ml. Constructed cosmid library in* E. coli* was stored in LB containing 15% (v/v) glycerol at −80°C.

### 2.3. DNA Manipulations

Total DNA from* L. raffinolactis* BGTRK10-1 was isolated by modified method described by Hopwood et al. [20]; the logarithmic phase cells were pretreated with lysozyme (4 mg/ml, for 15 min at 37°C) prior to treatment with SDS. For plasmid isolation from* E. coli* the QIAprep Spin Miniprep kit was used according to the manufacturer's recommendations (Qiagen, Hilden, Germany). Standard heat-shock transformation was used for plasmid transfer into* E. coli* [21]. Digestion with restriction enzymes was conducted according to the supplier's instructions (Thermo Fisher Scientific). The DNA fragments from agarose gels were purified using QIAqick Gel extraction kit as described by the manufacturer (Qiagen, Hilden, Germany). DNA was ligated with T4 DNA ligase (Agilent technologies, USA) according to the manufacturer's recommendations. Platinum™* Taq* DNA Polymerase High Fidelity (Thermo Fisher Scientific, Waltham, MA, USA) was used to amplify DNA fragments by PCR in GeneAmp PCR system 2700 thermal cycler (Applied Biosystems, Foster City, CA, USA). PCR products were purified with QiAquick PCR purification kit (Qiagen, Hilden, Germany) according to the manufacturer's recommendations. DNA sequencing was done by the Macrogen Sequencing Service (Macrogen Europe, The Netherlands). The primers used in PCR for amplification of LraI operon (*lraIR* and* lraIM *genes) were as follows: LraRM-Fw (5′-GTATAGAAAGAAGAATCG-3′) and LraRM-Rev (5′-GCAGGGTAATGTTCCTCAC-3′), while following primers were used for overexpression of* Lra*I restriction enzyme (*lraIR* gene) in pMALc5X vector: LraI-Fw (5′-ATGTCGAGAAAAAATCAGTCG-3′) and LraI-Rev (5′-CTCAAGCTTTCTAATTAATCCTTTTTTGC-3′;* Hin*dIII restriction site is underlined). Total DNA (1 ng) was mixed with 17.9 *μ*l of bidistilled water, 2.5 *μ*l of 10x PCR buffer (Thermo Fisher Scientific), 1 *μ*l dNTP mix (10 mM), 1.5 *μ*l of MgCl_2_ (25 mM), 1 *μ*l (10 pmol) of each primer, and 0.1 *μ*l of Platinum™* Taq* DNA Polymerase High Fidelity. Performed using the GeneAmp 2700 PCR Cycler (Applied Biosystems), the PCR programs consisted of initial denaturation (5 min at 96°C), 30 cycles of denaturation (30 s at 96°C), annealing (30 s at 40°C) and polymerization (2 or 1 min at 68°C), and an additional extension step of 5 min at 68°C. PCR fragments of LraI operon amplified using Platinum™* Taq* DNA Polymerase High Fidelity were cloned into pAZIL vector predigested with* Sma*I.

### 2.4. Recombinant* Lra*I Restriction Endonuclease Overexpression in* E. coli* and Purification

PCR fragment consisted of* lraIR* gene (from ATG to stop codon) obtained by LraI-Fw/LraI-Rev primers and Platinum™* Taq* DNA Polymerase High Fidelity was purified, digested with* Hin*dIII and cloned into pMAL-c5X vector digested with* Xmn*I and* Hin*dIII restriction enzymes and transformed into ER2523 competent cells (New England Biolabs, Ltd. UK) previously transformed with pAZIL-LraRM construct (Table 1). Transformants were selected on LA Petri dishes containing 2% glucose, ampicillin 100 *μ*g/ml, and erythromycin 300 *μ*g/ml at 23°C. Confirmation of fragment presence in adequate orientation was obtained by restriction enzyme analysis (with* Sac*I and* Hin*dIII digestion) and sequencing. Expression of recombinant protein was carried out at 23°C by induction with 0.1 mM isopropyl *β*-D-1-thiogalactopyranoside (IPTG). Purification (cell lysis, affinity chromatography, and cleavage of fusion protein with Xa protease) was performed according to manufacturer instruction (pMAL Protein Fusion & Purification System; New England Biolabs, Ltd., UK). Purified recombinant LraI restriction endonuclease was stored at −20°C in CM buffer (20 mM Tris-HCl pH7.4, 200 mM NaCl, 1 mM EDTA, and 1 mM DTT) containing 50% glycerol.

### 2.5. Endonuclease Assays

Endonuclease activity was assayed by incubating various amounts of purified* Lra*I enzyme in buffer recommended for use with* Eco*RI (50 mM Tris-HCl, pH 7.5, 10 mM magnesium chloride, 100 mM sodium chloride, 0.02% Triton X-100, supplemented with 100 *μ*g/ml BSA; Thermo Fisher Scientific) containing 1 *μ*g of pBluescipt SK+ plasmid DNA per 50 *μ*l reaction mixture for 1 h at 37°C. One unit of enzyme activity was defined as amount of purified* Lra*I enzyme, which was able to completely cut 1 *μ*g of plasmid DNA for 1 h.

Influence of reaction buffer composition on* Lra*I enzyme activity was assayed in different commercial buffers (Buffer B (blue; 10 mM Tris-HCl pH 7.5, 10 mM magnesium chloride, 100 *μ*g/ml BSA), Buffer G (green; 10 mM Tris-HCl pH 7.5, 10 mM magnesium chloride, 50 mM sodium chloride, 100 *μ*g/ml BSA), Buffer O (orange; 50 mM Tris-HCl pH 7.5, 10 mM magnesium chloride, 100 mM sodium chloride, 100 *μ*g/ml BSA), Buffer R (red: 10 mM Tris-HCl pH 8.5, 10 mM magnesium chloride, 100 mM potassium chloride, 100 *μ*g/ml BSA), Buffer Tango (yellow: 33 mM Tris–acetate pH 7.9, 10 mM magnesium acetate, 66 mM potassium acetate, 100 *μ*g/ml BSA), and buffer recommended for use with* Eco*RI; Thermo Fisher Scientific) used for reaction with 1 U of purified* Lra*I enzyme and 1 *μ*g of plasmid DNA for 1 h at 37°C. To measure the activity of purified* Lra*I enzyme at different temperatures 1 U of purified* Lra*I enzyme was incubated with 1 *μ*g of plasmid DNA for 1 h at different temperatures (13°C, 23°C, 30°C, 37°C, 45°C, 60°C, and 80°C). In all endonuclease activity assays commercial* Eco*RI restriction enzyme (Thermo Fisher Scientific) was used as control. Reactions were stopped by addition 1/10 volume of stop solution (50 mM EDTA pH 8, 50% glycerol, 0.02% orange G) and products were analyzed by electrophoresis in 1% agarose gels.

### 2.6. Determination of* Lra*I Cleavage Site

To determine the precise positions and nucleotide sequence of cleavage sites within double stranded DNA for the* Lra*I restriction enzyme, pBluescript SK+ was used as template. Plasmid was digested with recombinant enzyme* Lra*I; complete digestion was confirmed by agarose gel electrophoresis and digest was sequenced with M13F and M13R primers (Macrogen Europe, The Netherlands). Simultaneously as control, the whole experiment was conducted with commercial* Eco*RI restriction enzyme (Thermo Fisher Scientific).

### 2.7. Bioinformatic Analysis of* Lra*I Homologs

Sequence searches on the NCBI nucleotide and protein databases were conducted with BLAST [22] using* lraIR*/LraIR and* lraIM*/LraIM sequences. The phylogenetic inferences between restriction and methylase enzymes were obtained by MEGA version 6.0 (http://www.megasoftware.net/). The first 30 protein reference sequences of* Eco*RI-like endonuclease or methyltransferase enzymes chosen according to results of BLASTP search and LraI restrictase and LraI methylase sequences separately were trimmed and aligned using Clustal W [23] with default parameters. The phylogenetic trees were constructed by the maximum-likelihood (ML) method using a Tamura-Nei model. Bootstrapping of 1000 replicates was used to infer confidence levels of ML trees.

The nucleotide sequences of DNA fragments carrying genes encoding* Lra*I restriction-modification system and 16S rRNA from* L. raffinolactis* BGTRK10-1 were submitted to ENA GenBank under accession numbers LT222052 and LT854837, respectively.

## 3. Results and Discussion

### 3.1. Identification of* Lra*I (*Eco*RI-Like) Methylase Activity in* L. raffinolactis* BGTRK10-1

The mesophilic lactic acid bacterium* L. raffinolactis *is prevalent in dairy foods, such as raw milks, natural dairy starter cultures, and a great variety of cheeses.* L. raffinolactis* BGTRK10-1 is a natural isolate from autochthonous young sweet kajmak produced in the Vlašić mountain region of central Bosnia and Herzegovina [17]. Strain BGTRK10-1 was selected because of its strong autoaggregation phenotype. In order to construct cosmid library of strain BGTRK10-1 to clone aggregation ability coding gene(s), total DNA of the strain was digested with several restriction enzymes (including* Eco*RI). It has been observed that the* Eco*RI did not cut isolated DNA, in several attempts, unlike the other used restriction enzymes. It was suspected that the strain possesses RM system (named Lra * L. raffinolactis*) that recognizes the same DNA sequence as* Eco*RI RM system. Hence, the methylase activity of the strain* L. raffinolactis* BGTRK10-1, which protects its DNA from the digestion by* Eco*RI restriction enzyme, was quite accidentally discovered during routine laboratory work.

Restriction endonucleases, commonly known as restriction enzymes, are ubiquitously present in prokaryotes. The main function of restriction enzymes is the protection against foreign genetic material, especially against bacteriophage DNA. Several restriction-modification systems have been identified in lactococci. Most of them are plasmid encoded and function as phage-resistance mechanism, which is very important for the strains used in the dairy industry in terms of preventing phage infection and cell lysis [25–29].

### 3.2. Selection of Clone Carrying* Lra*I RM Operon from Cosmid Library

Cosmid DNA was isolated from total* Xba*I cosmid library in* E. coli* HB101 and 1 *μ*g of DNA mix from total cosmid clones was subjected to digestion with* Eco*RI restriction enzyme and after that directly transformed into DH5*α* competent cells. Cosmid DNA isolated from obtained transformants was rechecked for resistance to* Eco*RI restriction enzyme digestion. One cosmid, named pAZILcosLra, providing resistance to* Eco*RI restriction enzyme digestion was selected for further analyses: subcloning and DNA sequencing (Table 1).

### 3.3. LraI Operon for RM System Provides Resistance to* Eco*RI Restriction Enzyme Digestion

**Figure 1:**
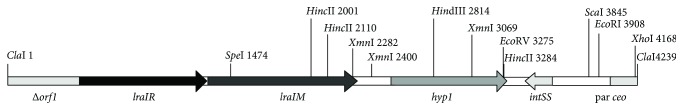
Schematic presentation of* Cla*I DNA fragment of 4239 bp carrying complete information for providing resistance to* Eco*RI digestion.* Cla*I DNA fragment containing following ORFs: truncated gene for pentapeptide repeat containing protein (Δ*orf1*),* Eco*RI-like endonuclease* (lraIR)*,* Eco*RI-like methylase* (lraIM)*, hypothetical protein* (hyp1)*, site specific integrase* (intSS)*, and partial gene for N(5)-(carboxyethyl) ornithine synthase (par* ceo*).

To localize the minimum genetic unit on the cosmid pAZILcosLra that is responsible for the resistance to digestion with* Eco*RI restriction enzyme, the cosmid pAZILcosLra was digested with several restriction enzymes (*Xba*I-generated four fragments,* Hin*dIII-three fragments,* Cla*I-four fragments, and* Eco*RV-three fragments) and then subcloned into pBluescript SK+ vector digested with corresponding restriction enzymes. Only one construct, pBSLraCla (obtained with* Cla*I), was able to reestablish the resistance to* Eco*RI restriction enzyme digestion (Table 1). The* Cla*I DNA fragment of 4239 bp carrying complete information for resistance to* Eco*RI digestion was completely sequenced by primer walking. Four complete (*Eco*RI-like endonuclease,* Eco*RI-like methylase, hypothetical protein, and site specific integrase), one truncated (pentapeptide repeat containing protein), and one partial (N(5)-(carboxyethyl) ornithine synthase) open reading frame (ORFs) were revealed on* Cla*I DNA fragment (Figure 1). Position of LraI RM operon in genome of strain BGTRK10-1 indicates the possibility that the operon was acquired by horizontal gene transfer; the conserved lactococcal gene for pentapeptide repeat containing protein is interrupted in the middle by LraI RM operon and immediately after methylase gene is located gene for site specific integrase. This event that occurred in the distant past is indicated by the fact that additional mutations were accumulated within the first part of the gene for pentapeptide repeat containing protein, most probably due to its nonfunctionality. The distance between the restrictase and the methylase genes is 10 nucleotides without promoter and ribosomal binding site and, in other EcoRI-like operons, strongly indicates polycistronic RNA transcription from upstream promoter and translation from consensus RBS (AGGAGA) 4 nucleotides distant from ATG codon of restrictase gene.

To confirm the functionality of* Lra*I restriction-modification operon, a region that includes both (*lraIR* and* lraIM*) genes was amplified using LraRM-Fwand LraRM-Rev primers (for details see Section 2.2) and cloned into pAZIL vectors giving construct pAZIL-LraRM. Construct carrying only these two genes was completely sequenced while resistance to* Eco*RI restriction enzyme digestion was confirmed* in vitro*.

### 3.4. Cloning, Overexpression, and Purification of* Lra*I Restriction Endonuclease

Plasmid clone pAZIL-LraRM was used as matrix for amplification of the open reading frame encoding* Lra*I restriction endonuclease with primers LraI-Fw and LraI-Rev. Since* Hin*dIII restriction site has been integrated into LraI-Rev primer, obtained amplified fragment was first treated with* Hin*dIII to provide directed cloning of PCR fragment into expression vector pMAL-c5X, which was digested with* Xmn*I and* Hin*dIII restriction enzymes. Ligation mix was transformed into ER2523 cells which were previously transformed with a pAZIL-LraRM vector expressing* Lra*I methylase, in order to protect transformed cells from the nuclease activity of* Lra*I towards their own. Transformants of ER2523/pAZIL-LraRM with pMAL-cX5LraI were successfully obtained when selection was carried out at 23°C on LA selective plates (erythromycin 300 *μ*g/ml and ampicillin 100 *μ*g/ml) containing 2% glucose in order to minimise expression of enzymes. Three clones (named pMAL-cX5LraI-29, pMAL-cX5LraI-31, and pMAL-cX5LraI-42, Table 1) were selected for restriction enzyme analysis, complete sequencing, and overexpression of enzyme.* Lra*I restriction nuclease was successfully overexpressed in all three clones by overnight induction with 0.1 mM IPTG at 23°C and purified using amylose resins and cleaved by Xa protease (which cleaves fusion protein between maltose binding protein and clone providing release of exactly the same protein as natural). The overexpression of* Lra*I restriction enzyme under aforementioned conditions (overnight induction with 0.1 mM IPTG at 23°C) represents the result that is similar to results observed by other researchers [30]. The possible explanation for this could be the expression of restriction enzymes is toxic at higher temperatures.

Purified* Lra*I restriction enzymes from all three clones were stored at −20°C in CM buffer with 50% glycerol.

### 3.5. Functional Analysis, Determination of Ionic Strength, and Temperature Optimum of the Purified* Lra*I Endonuclease Activity

**Figure 2:**
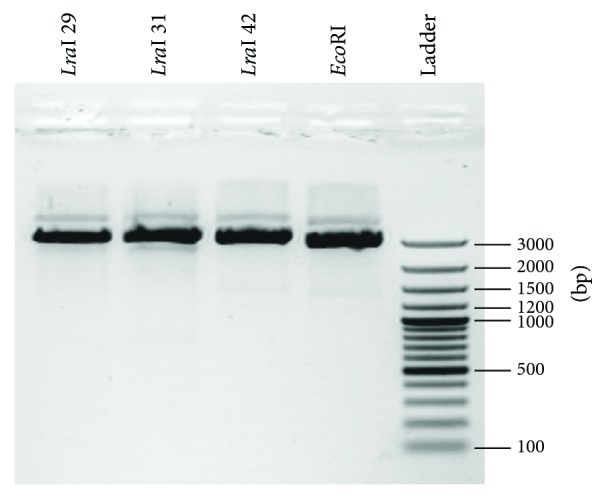
*Lra*I activity assay. Digestion of pBluescipt SK+ by purified* Lra*I restriction enzyme* Lra*I 29,* Lra*I 31, and* Lra*I 42 from clones pMAL-cX5LraI-29, pMAL-cX5LraI-31, and pMAL-cX5LraI-42, respectively.

Considering that* Lra*I RM system provided complete protection against digestion of* Eco*RI endonuclease, it was assumed that it recognizes and cleaves the identical nucleotide sequence. Since plasmid pBluescipt SK+ contains one* Eco*RI restriction site in polycloning region it was used for functional analysis of purified* Lra*I restriction enzyme. Specific activity (1 U) of purified* Lra*I restriction enzyme was determined in* Eco*RI reaction buffer (Thermo Fisher Scientific) at 37°C. Different levels of* Lra*I restriction enzyme expression were observed in selected clones, but specific activities (U/*μ*g of purified proteins) were almost the same (1 U/50 ± 5 ng) among the clones pMAL-cX5LraI-29, pMAL-cX5LraI-31, and pMAL-cX5LraI-42 (Figure 2).

**Figure 3:**
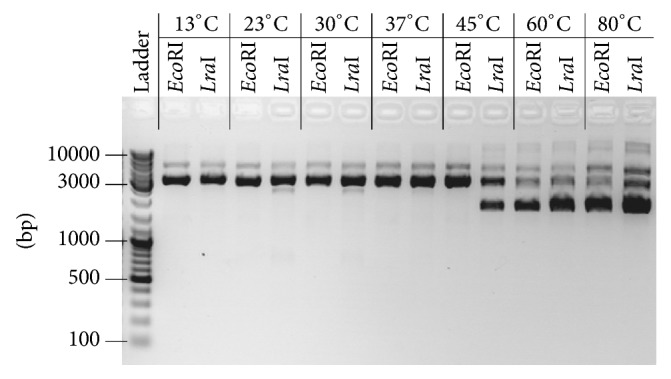
Determination of temperature optimum of the purified* Lra*I restriction enzyme activity. Commercial* Eco*RI restriction enzyme was used in control reactions.

To determine optimal temperature for* Lra*I activity, purified enzyme was incubated with pBluescipt SK+ vector at temperatures ranging from 13°C to 80°C.* Lra*I enzyme showed the highest activity at lower temperatures (between 13°C and 37°C), while at 45°C and higher temperatures it partially cut DNA (Figure 3). However, this is in agreement with the optimal growth temperature of strain BGTRK1-10 (30°C). Briefly, since the first description of* Eco*RI restriction enzyme in 1970 [31], more than 500 isoschizomers have been reported or predicted with very high levels of identity (50–70%) pointing to the widespread distribution among species of different Phyla and indicating possible common origin. It seems that some specific characteristics, such as optimum working temperature, diverged depending on the optimum growth temperature of the enzyme producing bacteria, which is why we think that* Lra*I exhibits better activity at lower temperatures. It was found that commercial* Eco*RI (used as control) showed high activity at 45°C in contrast to* Lra*I enzyme pointing to the difference between these two enzymes.

In addition, stability of* Lra*I enzyme was tested after different period of storage at −20°C;* Lra*I enzyme did not lose activity after storage for more than six months at −20°C in CM buffer with 50% glycerol.

**Figure 4:**
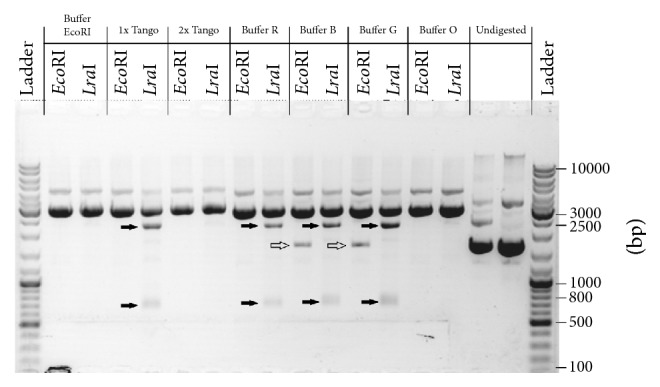
Determination of influence of ionic strength in different reaction buffers on the* Lra*I restriction enzyme activity. Commercial* Eco*RI restriction enzyme was used in control reactions. L: ladder (GeneRuler DNA Ladder Mix, Thermo Fisher Scientific). Black arrows present fragment obtained by* Lra*I*∗* activity; white arrows present undigested plasmid DNA in* Eco*RI (Thermo Fisher Scientific) digestion.

To establish the optimal salt concentration in the reaction buffer for* Lra*I enzyme activity different commercial buffers, Buffer B, Buffer G, Buffer O, Buffer R, Buffer Tango, and buffer recommended for use with* Eco*RI (Thermo Fisher Scientific) were used.* Lra*I enzyme exhibited high and specific activity in buffers with 100 mM and higher salt concentrations (Figure 4, buffer recommended for use with* Eco*RI, Buffer 2x Tango and Buffer O), except in Buffer R (red) (10 mM Tris-HCl pH 8.5, 10 mM magnesium chloride, 100 mM potassium chloride, and 100 *μ*g/ml BSA) (Figure 4; Buffer R). It was noticed that, in buffers with lower ionic strengths, the* Lra*I enzyme exhibited a specific star activity, cutting the vector at another position (Figure 4; Buffer 1x Tango, Buffer B, Buffer G).

It is interesting that in Buffer B and Buffer G commercial* Eco*RI restriction enzyme exhibited weaker activity, partial digestion.

### 3.6. *Lra*I Is an Isoschizomer of* Eco*RI

**Figure 5:**
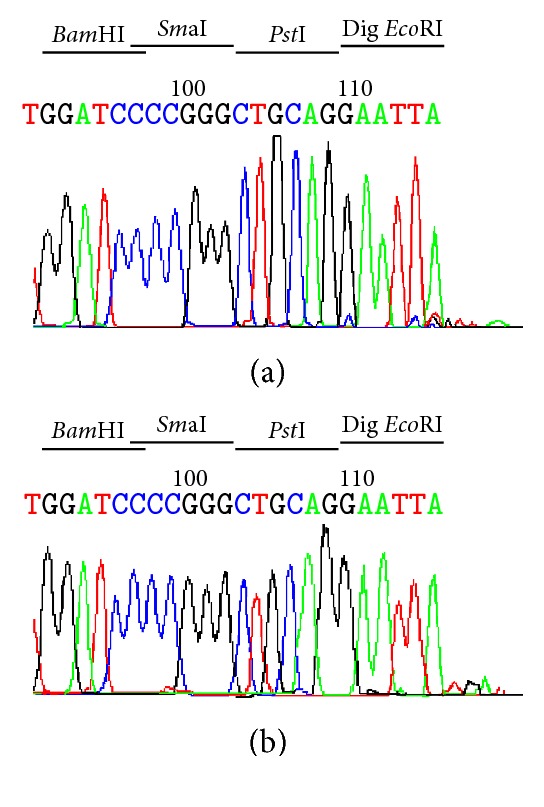
Determination of the* Lra*I cleavage site 5′-G/AATTC-3′ of pBluescipt SK+ by DNA sequencing. (a) Sequence of pBluescipt SK+ predigested with* Lra*I restriction enzyme and (b) with* Eco*RI obtained using M13R primer. The colour-coded sequence traces are A (green), T (red), C (blue), and G (black). The extra A base was added at the end of the cleaved template by the* Taq* DNA polymerase used for sequencing due to template-independent terminal nucleotide transferase activity of* Taq* DNA polymerase.

Sequencing of double stranded cleaved DNA by the* Lra*I enzyme has shown that the* Lra*I enzyme recognizes the identical nucleotide sequence (5′-G/AATTC-3′), as expected, and cuts it at the same position (between G and A) like* Eco*RI enzyme (Figure 5) leaving identical sticky ends. The same cleavage results were obtained with* Lra*I enzymes purified from all three clones which supported our conclusion that* Lra*I enzyme is an isoschizomer of* Eco*RI.

### 3.7. Determination of the Cleavage Site of* Lra*I*∗* Activity

One of the important characteristics of restriction enzymes is their high sequence specificity in order to adequately provide the function of protecting the genome integrity. In addition, it was established that restriction enzymes in nonoptimal conditions could exhibit a modified specificity so that the same restriction enzyme could recognize and cleave DNA at additional positions to canonical one [32]. For* Eco*RI restriction enzyme, it was detected that in at low ionic strength and high pH enzyme is transformed so that it recognizes and digests the shortened tetranucleotide sequence 5′-/AATT-3′ [32]. This activity is termed star activity and is usually labeled as “*∗*” adjacent to the enzyme name and was also detected for other restriction enzymes and could be used to cleave DNA at additional sites for cloning purposes. In order to be used for cloning star restriction enzyme activity should (i) be restricted to limited number of sequence variants, (ii) provide the same sticky ends for ligation into the vector, and (iii) be controlled by changeable conditions.

**Figure 6:**
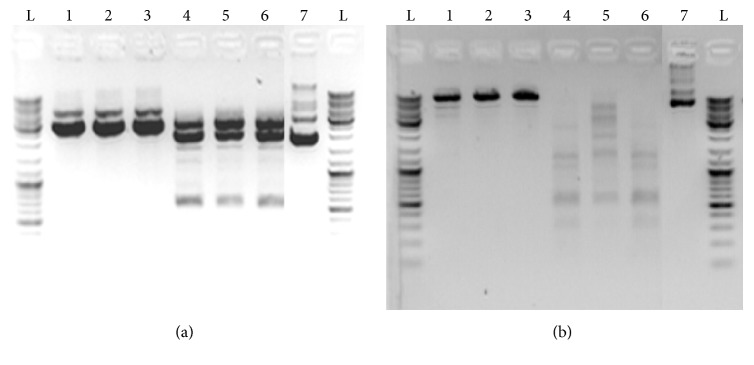
Determination of the cleavage site of* Lra*I*∗* activity. (a) Digestion of pBluescipt SK+ vector by* Lra*I 29 (1 and 4),* Lra*I 31 (2 and 5), and* Lra*I 42 (3 and 6) in buffer recommended for use with* Eco*RI (1, 2, and 3) and in 1x Tango Buffer (4, 5, and 6); 7: undigested pBluescipt SK+; digestion of pAZIL vector by* Lra*I 29 (1 and 4),* Lra*I 31 (2 and 5), and* Lra*I 42 (3 and 6) in buffer recommended for use with* Eco*RI (1, 2, and 3) and in 1x Tango Buffer (4, 5, and 6); 7: undigested pAZIL vector; L: ladder (GeneRuler DNA Ladder Mix, Thermo Fisher Scientific).

To determine the cleavage site of* Lra*I*∗* activity, the fragment of 672 bp obtained after digestion of pBluescipt SK+ in buffer with low ionic strength (1x Tango; Thermo Fisher Scientific) (Figure 6(a)) was cloned into pAZIL vector predigested with* Lra*I. Isolated plasmids (named pAZIL-LraI*∗*672pBS; Table 1) from selected white colonies were first checked for the presence of DNA fragment of 672 bp by restriction analysis with* Lra*I enzyme and then sequenced. The sequence of the entire fragment as well as the adjacent regions of the vector showed that the cloned 672 bp fragment originating from pBluescipt SK+ was cut out by* Lra*I*∗* enzyme activity at positions (701* Eco*RI) and at additional position (29) in which the 5′-AAATTC-3′ sequence is present. The same sequencing results were obtained for three independent clones confirming that additional recognition and cleavage site for* Lra*I*∗* activity is 5′-A/AATTC-3′ sequence. To be sure that only AAATTC sequence is recognized and cleaved by* Lra*I*∗* activity, but not other sequences with alternative changes within the GAATTC site, a search analysis for the presence of other alternatives in plasmid sequences was performed. Since all alternatives with one nucleotide change of recognition sequence exist in pBluescipt SK+ (in addition to AAATTC, at position 29, the following recognition sequences GAATTG, at position 647; CAATTC, at position 850; TAATTC, at position 2824 were found), but were not cleaved, we conclude that* Lra*I*∗* activity specifically recognizes and cleaves only one variant of degenerate recognition sequence (5′-A/AATTC-3′). To further test the conclusion obtained on pBluescipt SK+, the plasmid pAZIL sequence was analyzed and subjected to digestion by* Lra*I enzyme under conditions that induce star activity. The obtained digestion results were completely in correlation with the predicted expectations (nine positions/fragments) (Figure 6(b)), so we could conclude that* Lra*I*∗* activity is limited to only one variant of the recognition sequence giving identical cohesive ends as in optimal conditions making the resulting fragments after* Lra*I*∗* activity clonable without further processing into* Lra*I or* Eco*RI treated vectors.

*Lra*I restriction enzyme star activity meets all the given requirements: it recognizes only one variant of the sequence which can enable more precise restriction mapping and cloning, provides the same cohesive ends compatible with* Eco*RI (contained by most cloning vectors), and is completely controlled by low ion concentration and/or high pH. Both factors which induce star activity of* Lra*I restriction enzyme, low ionic strength (Buffer 1x Tango) and buffer with higher pH (Buffer R, pH 8.5), also influence on* Eco*RI, but, in contrast,* Lra*I*∗* recognizes only one additional sequence expressing more specific star activity.

### 3.8. Phylogenetic Similarity of* Lra*I Restriction-Modification Enzymes with Others Belonging to* Eco*RI-Like Group

A search for number of restriction enzymes recognizing 5′-GAATTC-3′ sequence present in REBASE revealed 526 putative* Eco*RI-like proteins. Protein BLAST analysis showed that 196 restriction enzymes in NCBI database (from various microorganisms) share more than 50% identity on at least 50% protein coverage with* Lra*I enzyme. Highest identity was observed with restriction endonucleases from streptococci (*Streptococcus suis* 68%,* Streptococcus dysgalactiae* 64%, and* Streptococcus mutans* 64%). It is interesting that similar but higher identity was observed for* Lra*I methylase, again with streptococci (*Streptococcus pseudopneumoniae *74%,* Streptococcus suis* 73%,* Streptococcus mutans* 73%, and* Streptococcus dysgalactiae* 68%). Similar percentage of identity was observed also at nucleotide level (about 70%) for both* Lra*I restrictase and methylase genes. The fact that most* Eco*RI isoschizomers, unlike other restriction enzymes, share a high level of identity, indicates their common origin [7].

**Figure 7:**
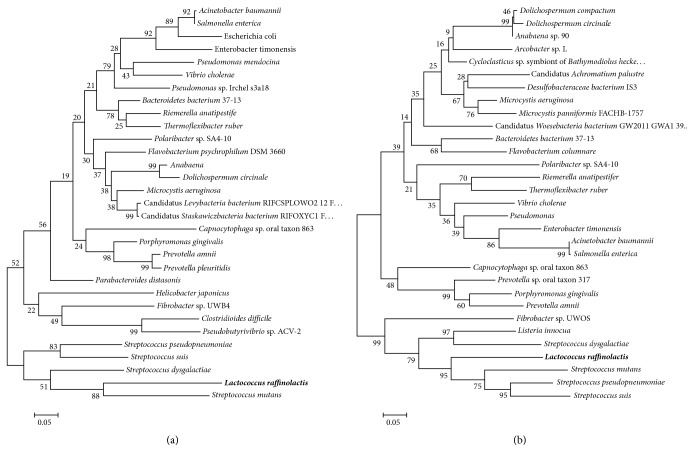
Phylogenetic similarity of* Lra*I RM system with other belonging to* Eco*RI-like group constructed using a Tamura-Nei model. (a) Phylogenetic tree for* Lra*I restrictase; (b) phylogenetic tree for* Lra*I methylase enzymes.

Phylogenetic trees were constructed for both* Lra*I restrictase (Figure 7(a)) and methylase (Figure 7(b)) enzymes. Phylogenetic analysis showed that* Lra*I homologs (restriction endonucleases and methylases) can be divided into two main branches, one (that could be additionally subdivided, comprising close homologs (Gram-negative bacteria and Cyanophyta) and the other comprising homologs from species belonging to Firmicutes phylum. The position of genus* Fibrobacter* (phylum: Fibrobacteres) which consists of only two species is interesting; its restrictase enzyme belongs to one branch, while methylase protein belongs to the other (Figure 7).

## 4. Conclusions

We identified a potent Type II restriction endonuclease in* L. raffinolactis* BGTRK10-1, named* Lra*I. The recognition and cleavage sequence for* Lra*I restriction enzyme was determined as 5′-G/AATTC-3′, indicating that* Lra*I restriction enzyme is an isoschizomer of* Eco*RI but with different characteristics. One of characteristics that has been thoroughly studied is star activity of* Lra*I restriction enzyme that is limited to one variant of the recognition site and cuts another alternative sequence 5′-A/AATTC-3′ leaving the same sticky ends on fragments as* Eco*RI, making the fragments obtained after digestion easy to clone without additional processing.

## Figures and Tables

**Table 1:** Bacterial strains and plasmids used in this study.

Strains or plasmids	Relevant characteristics	Source or reference
*Lactococcus raffinolactis*		
BGTRK10-1	Natural isolate from autochthonous sweet kajmak	[17]
*Escherichia coli*		
DH5*α*	*supE44 ΔlacU169 (*ø*80 lacZΔM15) hsdR17 recA1 endA1 gyrA96 thi-1 relA1*	[21]
HB101	F^−^ *hsdS20* (r_B_^−^ m_B_^−^) *supE44 recA13 ara-14 proA2 lacY1 rpsL20*(Sm^R^) *xyl-5 mtl-1 galK2 lacY1 λ*^−^	[24]
ER2523	*fhuA2 [lon] ompT gal sulA11 R(mcr- 73::miniTn10- -Tet* ^*S*^ *) 2 [dcm] R(zgb-210::Tn10- -Tet* ^*S*^ *) endA1 Δ(mcrC-mrr)114::IS10*	New England Biolabs, Ltd. UK
ER2523/pAZIL-LraRM	Competent cells obtained by transformation of ER2523 cells with pAZIL-LraRM	This study
Plasmids		
pAZIL	7109 bp, Em^r^, shuttle cloning vector	[19]
pAZIL-LraRM	PCR fragments of LraI operon from BGTRK10-1 cloned into pAZIL vector predigested with *Sma*I	This study
pAZIL-LraI*∗*672pBS	DNA fragment of 672 bp obtained after digestion of pBluescipt SK+ with *Lra*I*∗* activity cloned into pAZIL vector predigested with *Lra*I	This study
pAZILcos	8194 bp, Em^r^, shuttle cosmid vector	[19]
pAZILcosLra	Cosmid selected from total *Xba*I cosmid library of BGTRK10-1	This study
pBluescript SK+	2958 bp, Amp^r^, cloning vector	Stratagene
pBSLraCla	*Cla*I fragment of 4239 bp obtained from pAZILcosLra cloned into pBluescript SK+ vector	This study
pMAL-c5X	5677 bp, pMB1 origin, *lacI*, *malE*, *bla*, Factor Xa cleavage site;	New England Biolabs, Ltd. UK
pMAL-cX5LraI-29	PCR fragment of *Lra*I restriction endonuclease from pAZIL-LraRM cloned into pMAL-c5X vector predigested with *Xmn*I and *Hin*dIII restriction enzymes	This study
pMAL-cX5LraI-31	PCR fragment of *Lra*I restriction endonuclease from pAZIL-LraRM cloned into pMAL-c5X vector predigested with *Xmn*I and *Hin*dIII restriction enzymes	This study
pMAL-cX5LraI-42	PCR fragment of *Lra*I restriction endonuclease from pAZIL-LraRM cloned into pMAL-c5X vector predigested with *Xmn*I and *Hin*dIII restriction enzymes	This study
